# Graphitic Carbon Nitride for Photocatalytic Air Treatment

**DOI:** 10.3390/ma13133038

**Published:** 2020-07-07

**Authors:** Michal Baudys, Šárka Paušová, Petr Praus, Vlasta Brezová, Dana Dvoranová, Zuzana Barbieriková, Josef Krýsa

**Affiliations:** 1 Department of Inorganic Technology, University of Chemistry and Technology Prague, Technická 5, 166 28 Prague 6, Czech Republic; baudysm@vscht.cz (M.B.); sarka.pausova@vscht.cz (Š.P.); 2 Institute of Environmental Technology, VŠB-Technical University of Ostrava, 17. listopadu 2172/15, 708 00 Ostrava-Poruba, Czech Republic; petr.praus@vsb.cz; 3 Institute of Physical Chemistry and Chemical Physics, Faculty of Chemical and Food Technology, Slovak University of Technology in Bratislava, Radlinského 9, SK-812 37 Bratislava, Slovak Republic; vlasta.brezova@stuba.sk (V.B.); dana.dvoranova@stuba.sk (D.D.); zuzana.barbierikova@stuba.sk (Z.B.)

**Keywords:** g-C_3_N_4_, photocatalyst, gas phase, acetaldehyde, NO_x_

## Abstract

Graphitic carbon nitride (g-C_3_N_4_) is a conjugated polymer, which recently drew a lot of attention as a metal-free and UV and visible light responsive photocatalyst in the field of solar energy conversion and environmental remediation. This is due to its appealing electronic band structure, high physicochemical stability and earth-abundant nature. In the present work, bulk g-C_3_N_4_ was synthesized by thermal decomposition of melamine. This material was further exfoliated by thermal treatment. S-doped samples were prepared from thiourea or further treatment of exfoliated g-C_3_N_4_ by mesylchloride. Synthesized materials were applied for photocatalytic removal of air pollutants (acetaldehyde and NO_x_) according to the ISO 22197 and ISO 22197-1 methodology. The efficiency of acetaldehyde removal under UV irradiation was negligible for all g-C_3_N_4_ samples. This can be explained by the fact that g-C_3_N_4_ under irradiation does not directly form hydroxyl radicals, which are the primary oxidation species in acetaldehyde oxidation. It was proved by electron paramagnetic resonance (EPR) spectroscopy that the dominant species formed on the irradiated surface of g-C_3_N_4_ was the superoxide radical. Its production was responsible for a very high NO_x_ removal efficiency not only under UV irradiation (which was comparable with that of TiO_2_), but also under visible irradiation.

## 1. Introduction

Graphitic carbon nitride (g-C_3_N_4_) is a polymeric material based on melon units. The extraordinary diamond-like properties of g-C_3_N_4_ are thermal, chemical and photochemical stability due to heptazine-based building blocks and strong covalent bonds between carbon and nitrogen atoms. Another important property of g-C_3_N_4_ is its band gap energy of 2.7 eV, which enables absorption of visible light (λ ≤ 460 nm). This is why various applications of g-C_3_N_4_ in photochemistry, including photocatalysis, have been studied. Except PVD (physical vapour deposition), CVD (chemical vapour deposition) and synthetic procedures, g-C_3_N_4_ can be prepared by the thermal condensation of nitrogen-rich precursors, such as cyanamide, dicyandiamide, melamine, urea and so forth [1]. However, the photocatalytic applications of g-C_3_N_4_ are limited by fast recombination of photoinduced electrons and holes, which causes low quantum efficiency. It can be overcome by doping with metals and non-metals. Doping with non-metals, such as phosphorus, sulphur, oxygen, boron, carbon, nitrogen and halogens, leads furthermore to a red shift of the absorption edge [2].

One of the promising applications of semiconductor photocatalysis is improvement of air quality in interiors. Aldehydes and other VOC (volatile organic compounds), which are released by furniture, may cause the sick building syndrome including non-specific diseases, e.g., respiratory irritation symptoms, headaches, dizziness, and fatigue [3]. Removal of acetaldehyde and formaldehyde is also part of the ISO 22197 methodology for evaluating the photocatalytic efficiency of pollutant removal from the gaseous phase [4]. The mechanism of photocatalytic removal of formaldehyde is often explained by the attack of a HO^∙^ radical to form an acetyl radical, followed by a subsequent radical reaction to generate the final products CO_2_ and formaldehyde [5]. Formaldehyde can be further photocatalytically oxidized to CO_2_ and H_2_O via a mechanism described in [6].

CH_3_CHO + HO^∙^ → CH_3_CO^∙^ + H_2_O (1)

CH_3_CO^∙^ + ½O_2_ → CH_3_COO^∙^ (2)

CH_3_COO^∙^ → ^∙^CH_3_ + CO_2_ (3)

^∙^CH_3_ + O_2_ → CH_3_COO^∙^ (4)

CH_3_COO^∙^ + HO^∙^ → HCHO + ½O_2_ + H_2_O (5)

Another application of photocatalytic processes in air treatment is the removal of NO_x_. This process is based on the oxidation of NO and NO_2_ (NO_x_) in the gaseous phase into NO_2_^−^/NO_3_^−^ in the aqueous phase. This process is also used as a standard ISO method for the determination of the photocatalytic efficiency of NO_x_ removal (ISO 22197-1) [6,7]. In the case of TiO_2_ as photocatalyst, the photocatalytic oxidation of NO in the inlet gaseous stream proceeds according to reactions (6–7). Potential accumulation of nitric acid on the photocatalyst surface may cause generation of NO_2_ (according to reaction 8), which is more toxic than NO. This can eventually lead to a steady state where the rate of NO decomposition is matched by the rate of NO_2_ production. It follows that nitric acid must be periodically washed out (e.g., by rain), to minimise the NO_2_ production.

2 NO + ½ O_2_ + H_2_O (TiO_2_, hν) → 2 HNO_2_ (6)

2 HNO_2_ + O_2_ (TiO_2_, hν) → 2 HNO_3_ (7)

2 HNO_3_ + NO (TiO_2_, hν) → 3 NO_2_ + H_2_O (8)

In a previous work, Praus et al. [8] developed a novel procedure based on the post-synthetic derivatization of g-C_3_N_4_ with methanesulfonyl (mesyl (CH_3_SO_2_^−^) chloride) and thus synthetized S-doped g-C_3_N_4_ and exfoliated g-C_3_N_4._ The obtained nanomaterials were characterized in detail but their photocatalytic activity was tested only in the liquid phase and by means of the decomposition of Acid Orange 7 under ultraviolet A (UVA) irradiation. 

Therefore, the aim of the present work was the evaluation of the influence of exfoliation and doping on the photoactivity of g-C_3_N_4_ in the gaseous phase using both UVA and VIS light. As model gaseous pollutants, acetaldehyde and NO were selected and the results were compared with the TiO_2_ photocatalyst (P25), which is considered as a benchmark for practical applications.

## 2. Experimental

### 2.1. Sample Preparation and Characterization

Bulk g-C_3_N_4_ (CN) was prepared by thermal treatment of melamine at 550 °C and further exfoliated at 500 °C using a procedure described in [9] (sample marked Ex-CN). In this way, the specific surface area increased from 8 to 93 m^2^ g^−1^. Sulphur-doped g-C_3_N_4_ photocatalysts were prepared by (i) thermal treatment of thiourea (sample S-CN) and (ii) derivatization of previously prepared bulk and exfoliated g-C_3_N_4_ with mesylchloride (sample marked Mes-Ex-CN). The procedure is described in ref. [8].

Bulk, exfoliated and sulphur-containing C_3_N_4_ structures had previously been characterized by various techniques (XRD, UV-VIS DRS, FTIR, BET, SEM, TEM, XPS [8], etc.).

For comparison, two TiO_2_ photocatalysts, namely the well-established P25 (Evonik) and pure anatase CG100 (Precheza) of comparable BET surface area as g-C_3_N_4_, were involved in the study (Table 1).

The immobilization of the synthetized g-C_3_N_4_ powder and commercial TiO_2_ materials on glass substrates was performed by drop casting from suspension and drying. The amount of photocatalytic material has been kept constant as 0.5 mg/cm^2^.

### 2.2. Photodegradation Process Setup—NO and Acetaldehyde

The efficiency of the photocatalytic removal of NO and acetaldehyde (AcAh) from the gaseous phase was measured in a single-pass flow-through reactor according to standard ISO methods (ISO 22197-1 removal of NO_x_ [4] and ISO 22197-2 removal of acetaldehyde [7]). The use of ISO methods is essential for possible comparison of results from different laboratories. An overview of ISO methods in photocatalysis was given recently by Mills et al. [6]. Defined process parameters (input gas concentration, flow rate, humidity, radiation source, etc.) are listed in Table 2. The content of g-C_3_N_4_ in all immobilized photocatalyst layers was fixed (0.5 mg/cm^2^).

**Figure 1:**
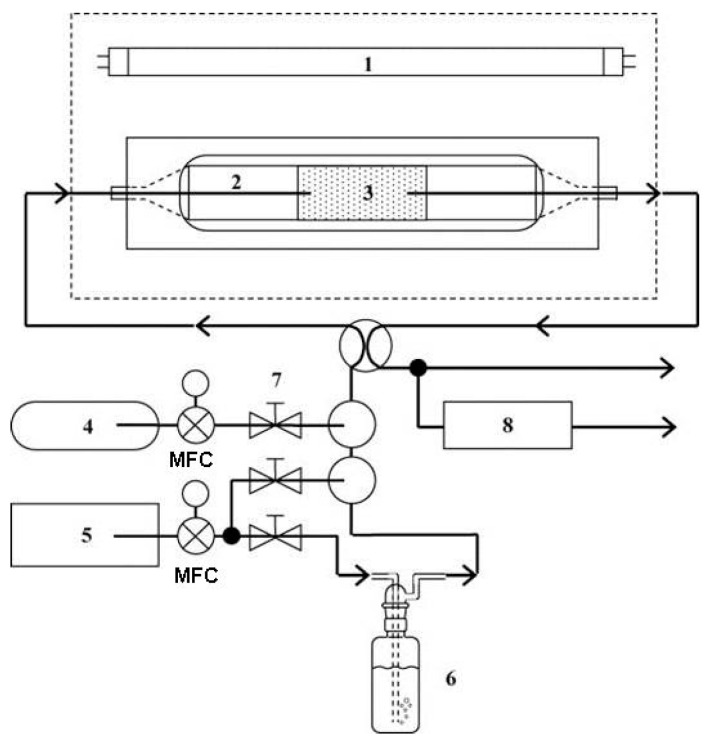
Apparatus for the evaluation of photocatalytic activity in the gas phase. 1: black light tube, 2: reactor, 3: catalyst, 4: pollutant gas, 5: compressed (synthetic) air, MFC: mass flow controller, 6: bubbler for humidification, 7: valves, 8: analyzer. Reprinted with permission from [10].

As pollutant sources, calibrated gas mixtures from SIAD (Rajhradice, Czech Republic) were used: 100 ppm NO in N_2_ and 250 ppm acetaldehyde in N_2_. These mixtures were further diluted with humidified air using mass flow controllers (see Figure 1). The final mixture, as admitted to the reactor, was either 5 ppm acetaldehyde or 1 ppm NO in air. The relative humidity (RH) was set to 50%. Analysis of the gaseous mixture was performed by a chemiluminescence analyzer (NO and NO_2_) and a gas chromatograph equipped with a flame ionization detector (GC-FID) in case of acetaldehyde.

**Figure 2:**
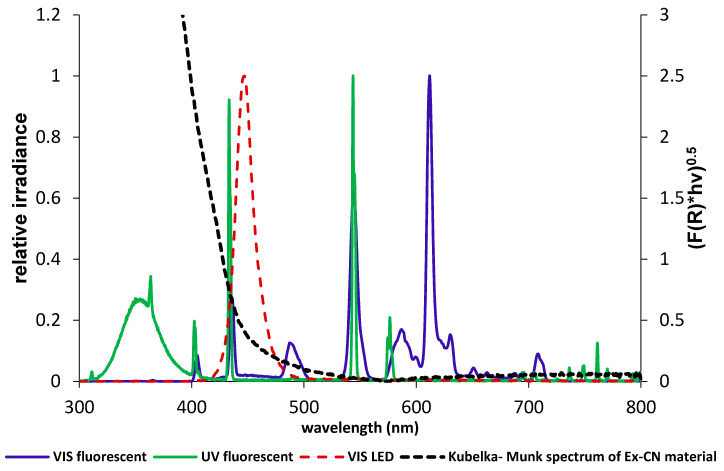
Emission spectra (left axis) of used light sources, UV (fluorescent), VIS (LED), VIS (fluorescent tube) and Kubelka–Munk spectrum of Ex-CN material (right axis).

The active part of the reactor (photocatalyst-coated plate) was 5 cm × 10 cm and the distance from the glass cover was 0.5 cm. The emission spectra of the incident light for all light sources at the location of the reactor were measured using a spectroradiometer (Specbos 1211, Jeti, Germany) and are shown in Figure 2. As a UV light source, a 25 W blacklight (UVA) tubular light source (351 nm centre wavelength) was used. The irradiance at the location of the reactor was 1 mW/cm^2^ between 340 and 400 nm. 

As a VIS light source, either an LED array (450 nm centre wavelength, irradiance 4 mW/cm^2^ in the wavelength range of 400–480 nm) or a fluorescent tube in combination with a liquid UV filter (1 M NaNO_2_ enabling transmission of light with λ > 400 nm), with an irradiance of 0.4 mW/cm^2^ between 400 and 480 nm, was used.

### 2.3. EPR Measurements

The X-band electron paramagnetic resonace (EPR) spectra of powder samples were monitored at room temperature by an EMX*Plus* EPR spectrometer (Bruker, Rheinstetten, Germany) operating at 100 kHz field modulation using a High Sensitivity Probe-head (Bruker). The *g*-factors were determined with an uncertainty of ± 0.0005 exploiting a nuclear magnetic resonance teslameter (ER 036TM, Bruker, Rheinstetten, Germany) and integrated frequency counter. The samples were placed in thin-walled quartz EPR tubes (Bruker) and the spectra were monitored in dark and upon continuous in situ irradiation using an UV LED source (*λ*_max_ = 365 nm; Bluepoint LED, Hönle UV Technology, Gräfelfing/München, Germany; irradiance of 15 mW cm^−2^ within the EPR cavity determined by a UVX radiometer (UVP, Upland, CA, USA)) or using a visible-light source (KL 1600LED (T = 5600 K); Schott, Mainz, Germany). Standard EPR spectrometer settings were as follows: microwave frequency, 9.426 GHz; microwave power, 10.66 mW; center field, 336 mT; gain, 2.52 × 10^5^; modulation amplitude, 0.1 mT; sweep time, 41 s; time constant, 20.48 ms; number of scans, 10. The generation of oxygen-containing reactive radicals upon in situ LED@365 nm exposure of aerated aqueous suspensions of photocatalysts was studied using the EPR spin-trapping technique with 5,5-dimethyl-1-pyrroline *N*-oxide (DMPO, Sigma-Aldrich, Buchs, Switzerland, distilled prior to the application). The spin-trapping experiments were performed at least in triplicate and the experimental spectra were processed and analyzed analogously as described in [11]. The spin-Hamiltonian parameters and relative concentration of individual DMPO-adducts were calculated using the EasySpin program [12].

## 3. Results

According to XRD phase analysis, TiO_2_ P25 contains mostly the anatase phase (70%) with a crystal size around 22 nm. The remaining phase is rutile with a similar crystal size. The second commercial TiO_2_ marked CG100 contains only the anatase phase with a smaller crystal size (12 nm), which corresponds to a higher surface area (70–110 m^2^/g) compared to TiO_2_ P25 (45 m^2^/g). 

In the case of g-C_3_N_4_ materials, two diffraction patterns corresponding to a CN hexagonal phase were observed. (the diffractogram is shown as Figure 3 in ref. [8]). Compared to TiO_2_, the crystal size of the g-C_3_N_4_ material is much lower (about 6 nm, detailed information given in [8]). The crystal size after exfoliation does not change. This observation is not in agreement with the specific surface area obtained by BET, which significantly differs between bulk material (CN) and exfoliated material (Ex-CN), (see Table 1). This can be explained by partial delamination of g-C_3_N_4_ particles providing non-diffracting nanosheets.

Prepared materials were characterized using the FTIR–ATR technique. Spectra are shown in Figure S1 (Supplementary Information). The broad weak spectral band in region (3300–3000 cm^−1^) can be assigned to stretching vibrations of N-H bonds. Other bands in region 1630–1232 cm^−1^ correspond to stretching vibration of C=N, and a C-N heterocyclic ring. The band at 804 cm^−1^ is attributed to the breathing vibration of triazine units.

Photoelectron spectroscopy was used to prove the presence of carbon, oxygen and sulfur. An example of an S 2p spectrum is shown in Figure S2. Two types of incorporated sulfur were indicated. A first binding energy around 164 eV indicates sulfur in a low oxidation state (sulfide or thiol). The second couple of 2p binding energies corresponds to sulfur in a lower oxidation state (mesyl groups). A detailed description of XPS and FTIR spectra of g-C_3_N_4_ has been reported previously [8].

For the C_3_N_4_ materials, the calculated band gap energies vary from the lowest values (2.63 eV) observed for S-CN, to 2.77 eV observed for Ex-CN. From that, it follows that the excitation wavelength must be lower than 471 nm (S-CN) and 448 nm (Ex-CN), respectively [8]. As it is well known, the band gap energy for anatase is 3.2 eV and for rutile 3.0 eV, which means that anatase absorbs only UV light (lower than 388 nm), while rutile can absorb also a small part of visible light (lower than 414 nm). From the emission spectra of VIS fluorescent tubes (Figure 2), it follows that C_3_N_4_ materials can utilize both peaks (404 and 435 nm) for excitation of electrons, while TiO_2_ P25 can utilize only one peak (403 nm) due to the presence of the rutile phase.

### 3.1. Acetaldehyde Removal

**Figure 3:**
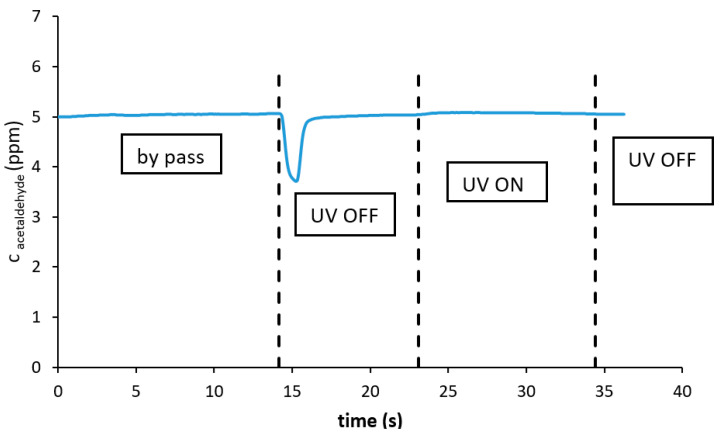
Removal of acetaldehyde on a film of Ex-CN (0.5 mg/cm^2^).

The concentration of acetaldehyde as a function of time at the output of the flow through system during the test for sample Ex-CN is shown in Figure 3. After reaching a stable input concentration of AcAh, the test gas was introduced to the reactor chamber in the dark, resulting in a decrease in concentration caused by adsorption. After reaching the adsorption equilibrium, the initial concentration was restored. After switching the UV light on, no decrease in the AcAh concentration was observed, which indicates that the exfoliated sample (Ex-CN) did not show a measurable conversion of acetaldehyde. Similar results were obtained for S-CN and Mes-Ex-CN.

**Figure 4:**
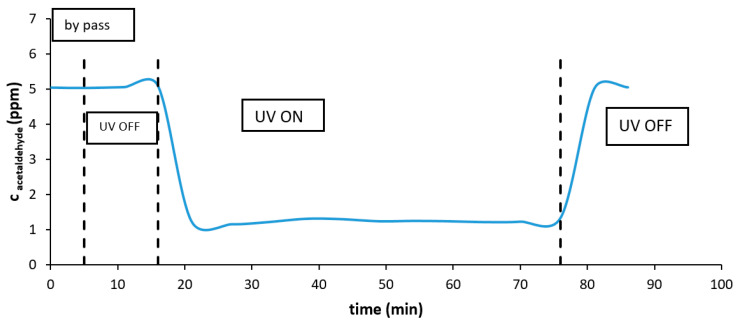
Removal of acetaldehyde on a film of TiO_2_ P25 (0.5 mg/cm^2^).

Figure 4 shows the removal of acetaldehyde for an immobilized TiO_2_ P25 layer. We can see a significant decrease in the acetaldehyde concentration after the UV light is switched on. The conversion of acetaldehyde on the TiO_2_ layer was about 80%.

### 3.2. NO_x_ Removal

**Figure 5:**
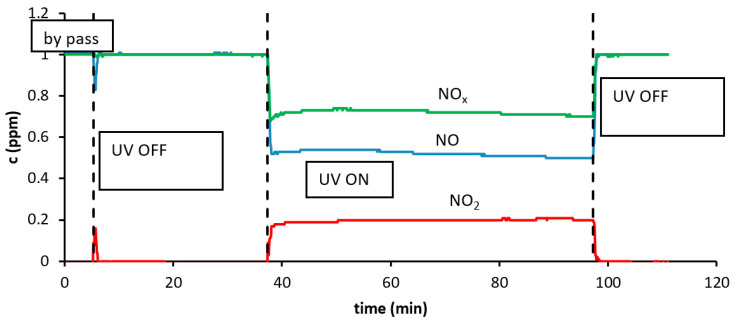
Removal of NO_x_ on a film of Ex-CN (0.5 mg/cm^2^).

The concentration of NO, produced NO_2_ and the sum of NO and NO_2_ (NO_x_) as a function of time for an immobilized layer of Ex-CN is shown in Figure 5. In contrast to acetaldehyde, after switching the UV light on, the decrease in the NO concentration immediately set in (to 0.5 ppm) accompanied by an increase in the NO_2_ concentration (to 0.2 ppm). These concentrations remained fairly constant during irradiation. It can be seen that the conversion of NO was about 50% and the conversion of NO_x_ was about 30%.

**Figure 6:**
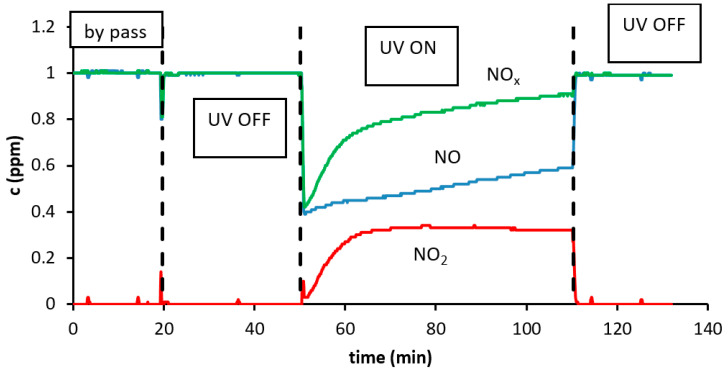
Removal of NO_x_ on a film of TiO_2_ P25 (0.5 mg/cm^2^).

The dependence of NO, NO_2_ and NO_x_ concentration is shown for an immobilized layer of TiO_2_ P25 in Figure 6. We can see a similar trend, only the increase in the NO_2_ concentration was slower but the constant value reached after 1 h of irradiation was 0.2 ppm and the concentration of NO achieved was 0.5 ppm.

**Figure 7:**
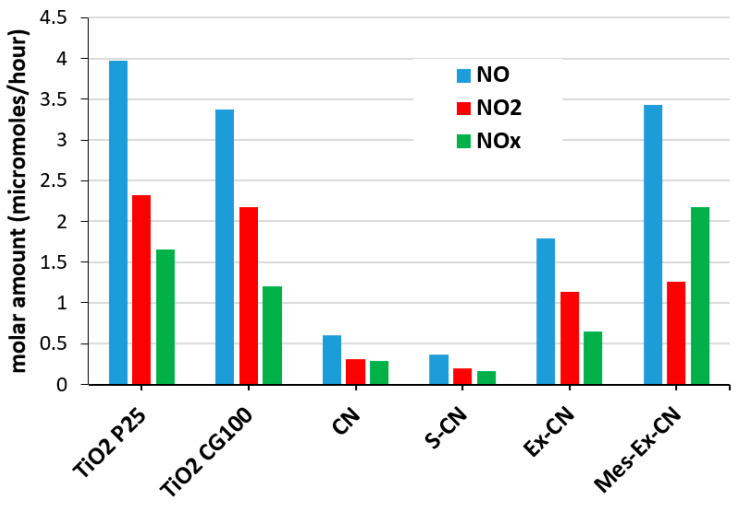
Amount of removed NO, formed NO_2_ and totally removed NO_x_, respectively, under UV irradiation for 1 h.

The amounts of removed NO, produced NO_2_ and removed NO_x_, respectively, under UV irradiation for various photocatalysts are summarized in Figure 7. In the case of bulk material (CN), the removal rate of NO_x_ is only about 0.3 µmol/h. An even lower removal rate was observed for a material prepared from thiourea (S-CN). An increase in the removal rate of NO_x_ was observed for exfoliated C_3_N_4_ (Ex-CN) (0.7 µmol/h), but this is still only half of the NO_x_ removal rate for P25 TiO_2._ Significant improvement in the removed NO_x_ can be seen for an exfoliated sample treated using mesyl chloride (Mes-Ex-CN). Although the amount of removed NO was lower than that for P25 TiO_2_, the amount of produced NO_2_ was two times lower for C_3_N_4_, resulting in a higher overall NO_x_ removal capability. In comparison with pure anatase (CG100) an even more significant improvement in the removal rate of NO_x_ (two times) was observed, mainly due to the lower NO_2_ production.

**Figure 8:**
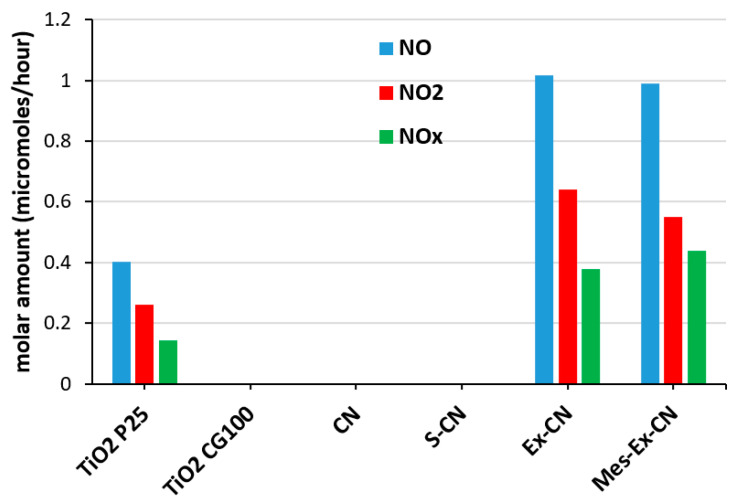
Amount of removed NO, NO_x_ and formed NO_2_, respectively, under VIS fluorescent irradiation during a 1 h NO_x_ experiment.

Figure 8 shows a comparison of the ability of various photocatalysts to remove NO and NO_x_ leading to the production of NO_2_ under VIS irradiation by fluorescent tubes. As expected, the TiO_2_ CG100 photocatalyst containing pure anatase did not show measurable photocatalytic activity using VIS light. On the other hand, TiO_2_ P25 containing about 30% rutile (estimated by XRD) showed a small but still measurable conversion of NO_x_ due to the presence of light at the wavelength of 405 nm. In the case of C_3_N_4_ materials, measurable NO_x_ conversion was obtained for exfoliated sample only (Ex-CN and Mes-Ex-CN), which showed similar activity. Bulk (non-exfoliated) samples (CN and S-CN) showed small NO_x_ conversion under UV light. Under VIS light, no measurable conversion of NO_x_ was observed. 

**Figure 9:**
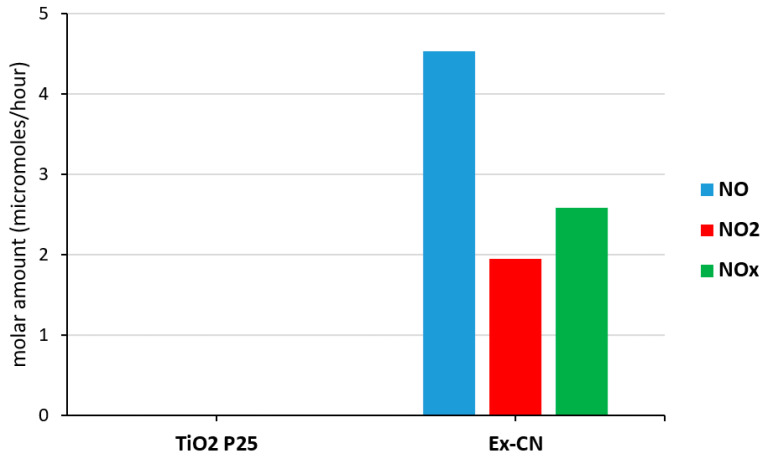
Amount of removed NO, NO_x_ and formed NO_2_ under VIS LED irradiation during 1 h of experiment.

In the next step, a monochromatic LED light source, which had a maximal intensity at 450 nm (which is closer to the C_3_N_4_ band gap threshold), was used to assess the photocatalytic activity in the visible light. The exfoliated sample (Ex-CN) and TiO_2_ P25 were compared and results are shown in Figure 9. Due to the absence of photons below 420 nm (Figure 2), TiO_2_ P25 showed no NO_x_ conversion. In the case of Ex-CN material, we can see a high level of NO_x_ removal, which is approximately six times higher compared to the NO_x_ removal in Figure 8. It should be mentioned that the VIS fluorescent source and VIS LED source differ dramatically in intensities. In the case of the VIS LED, it was 10 times higher so that the increase in NO_x_ removal was proportional to the increase in light intensity.

The C_3_N_4_-based nanomaterials usually possess paramagnetic centers traceable by EPR spectroscopy, which reflect the applied synthetic procedure and post-synthetic treatments. All the studied nanostructures exhibited the presence of an EPR signal prior to the photoexcitation and the EPR spectra obtained at room temperature (RT) represent a single Lorenztian-line with *g* = 2.0038. An analogous signal was monitored previously in C_3_N_4_-based nanomaterials [13,14] and was assigned to the unpaired electrons in the aromatic rings of carbon atoms in the localized π-states of typical heptazine.

**Figure 10:**
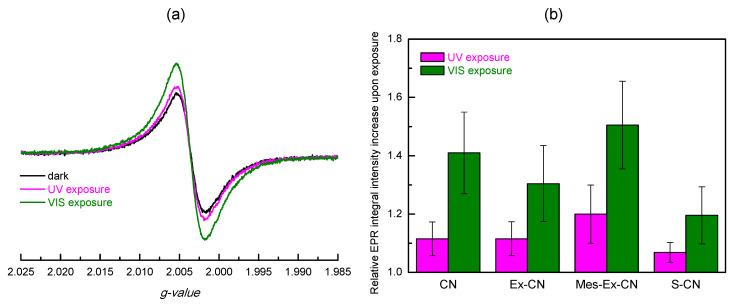
(**a**) EPR spectra obtained before and upon continuous ultraviolet A (UVA) (*λ*_max_ = 365 nm) and VIS (*λ* > 420 nm) exposure of the CN powder sample at room temperature. (**b**) Relative EPR integral intensity increase monitored upon UVA and VIS exposure of the studied powder samples at room temperature.

The performance of the studied nanomaterials upon continuous exposure was also followed by EPR, as the photogenerated charge carriers and their behaviour are coupled with the emergence of paramagnetic species. The UVA (*λ*_max_ = 365 nm) as well as visible-light (*λ* > 420 nm) exposure of the studied nanomaterials led to an increase in the intensity of the Lorentzian single line at *g* = 2.0038, while a more prominent effect was induced by visible-light exposure (Figure 10a). The increase in the intensity of the Lorentzian signal monitored upon photoactivation reflects the process of charge carrier formation/trapping. The signal is ascribed to the electrons in the localized states, especially the surface-trapped photogenerated electrons with preferentially carbon character, and the missing hyperfine interaction of nitrogen nuclei is attributed to the EPR line broadening [14,15]. To compare the effect of the light exposure on all the studied nanomaterials, the ratio of EPR integral intensity elucidated from the double-integrated X-band EPR spectra of the samples measured at RT upon UVA or VIS-light exposure and in the dark was calculated, providing the values of the relative EPR integral intensity increase presented in Figure 10.

### 3.3. Mechanism of Photocatalytic Removal

**Figure 11:**
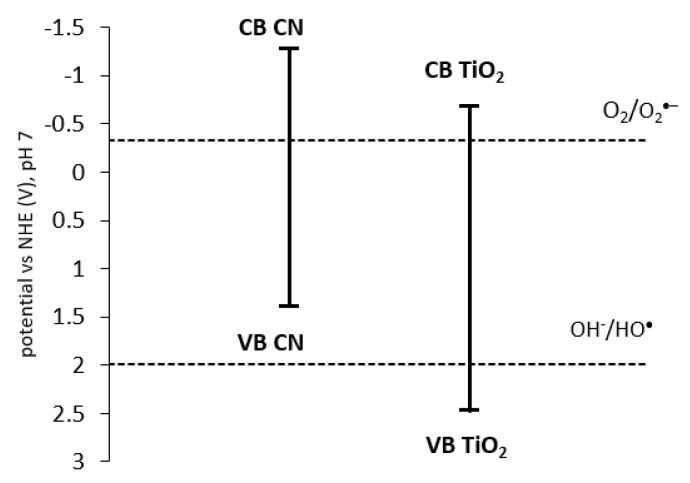
Position of valence-band and conduction-band edge for g-C_3_N_4_ and TiO_2_ at pH 7 [17].

The different efficiencies of photocatalytic removal of NO_x_ and acetaldehyde on the C_3_N_4_ and TiO_2_ photocatalyst can be explained by the particulate positions of their valence-band (VB) and conduction-band (CB) edges. The position of the valence-band edge (VB_edge_) of C_3_N_4_ is only 1.4 V (vs. NHE, pH 7) [16], while for the formation of hydroxyl radical a more positive potential (about 2 V vs. NHE, pH 7) is needed (Figure 11) [17]. This means that the oxidation potential of h^+^ in the VB of C_3_N_4_ is not sufficient to produce hydroxyl radicals according to Equation (9) (as in the case of TiO_2_ [18]), which are considered a primary species in the oxidation of acetaldehyde [19].

In ref. [20], the authors reported the position of the VB (1.57 V vs. NHE) and E_g_ (2.7 eV) for g-C_3_N_4_ materials. After hydrothermal delamination, VB shifted to 1.27 V vs NHE and E_g_ decreased to 2.3 eV. Thus, the position of VB and band gap energy depends on the preparation procedure and the subsequent treatment).

h^+^ + H_2_O → HO^∙^ + H^+^ (9)

As was discussed above, although irradiated C_3_N_4_ does not produce HO^∙^ radicals, we observed a significant NO_x_ conversion. Consequently, under the given experimental conditions, NO and HNO_2_ cannot be oxidized by HO^∙^ radicals (Equations (6) and (7)), as is the case for TiO_2_. Most probably, other oxygen-containing radical species are involved in the oxidation pathway. The CB-edge of C_3_N_4_ is more negative compared to TiO_2_, which predestines strong reduction properties of photogenerated e^−^. In the presence of molecular oxygen, the photoproduced electrons are readily scavenged, forming the superoxide radical anion (Equation (10)) [21].

**Figure 12:**
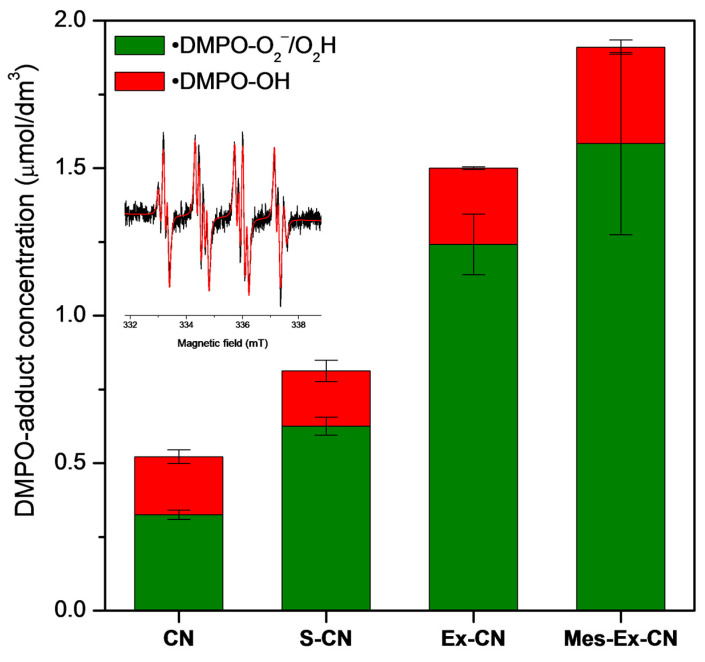
Concentration of ^∙^DMPO-O_2_/O_2_H and ^∙^DMPO-OH spin-adducts evaluated from the EPR spectra measured upon UV exposure of aerated aqueous suspensions of photocatalysts in the presence of 5,5-dimethyl-1-pyrroline *N*-oxide (DMPO) spin trap. (*λ*_max_ = 365 nm, UVA dose 4.5 J/cm^2^, photocatalyst loading, 0.5 mg/mL; *c*_0_(DMPO) = 0.04 mol/dm^3^). Inset: experimental spectrum (black line) obtained upon continuous irradiation of a Mes-Ex-CN suspension under the given experimental conditions along with its simulation (red line).

**Scheme 1:**
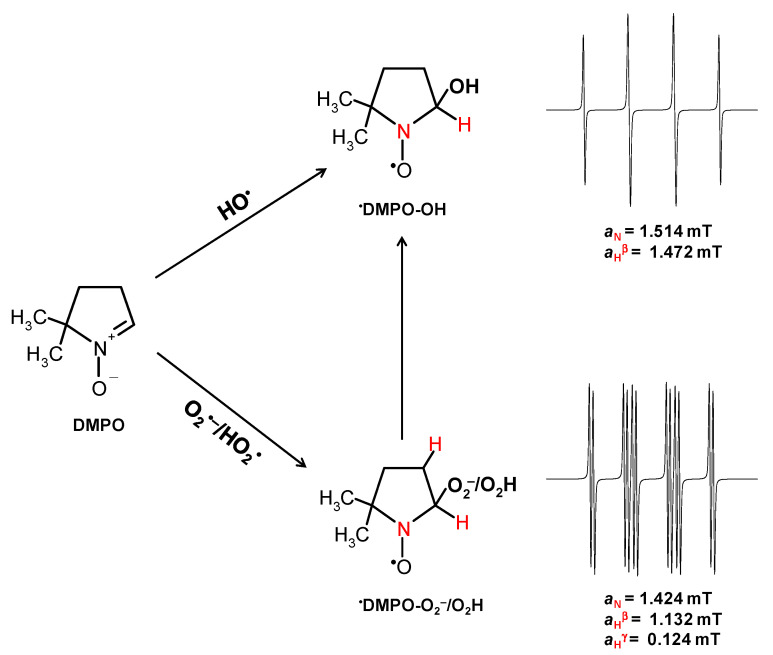
Illustration of DMPO spin-adducts generation upon UV exposure of aerated aqueous suspensions of C_3_N_4_-based photocatalysts along with simulated EPR spectra (magnetic field sweep of 7 mT).

The EPR spin-trapping technique was applied to evidence the generation of O_2_^∙^^−^/HO_2_^∙^ in the aerated aqueous suspension of photocatalysts. Figure 12 illustrates the ability of various types of C_3_N_4_ materials irradiated by UV light to produce DMPO spin-adducts. For all dispersed C_3_N_4_ photocatalysts, the monitored EPR spectra (inset in Figure 12) represent the superposition of two spin-adducts, i.e., the dominant twelve-line signal of ^∙^DMPO-O_2_/O_2_H (*a*_N_ = 1.424 ± 0.002 mT, *a*_H_^β^ = 1.132 ± 0.003 mT, *a*_H_^γ^ = 0.124 ± 0.002 mT; *g* = 2.0058 ± 0.0001) and a four-line signal (*a*_N_ = 1.514 ± 0.005 mT, *a*_H_^β^ = 1.472 ± 0.006 mT; *g* = 2.0057 ± 0.0001) characteristic for the ^∙^DMPO-OH adduct. The presence of ^∙^DMPO-O_2_/O_2_H unambiguously evidences the UV-light-induced generation of O_2_^∙^^−^/HO_2_^∙^ in the photocatalytic system. Accepting the fact that the hydroxyl radicals are not produced via oxidation by holes, (Equation (9)), the ^∙^DMPO-OH spin-adduct may originate from different sources (e.g., hydroxyl radicals from hydrogen peroxide photo/photocatalytic reactions, as dismutation of O_2_^∙^^−^/HO_2_^∙^ in water produces H_2_O_2_ [22], or by the ^∙^DMPO-O_2_/O_2_H transformation in aqueous media [23]). Scheme 1 illustrates the generation of both DMPO spin-adducts along with their simulated EPR spectra.

The concentration of ^∙^DMPO-O_2_/O_2_H followed by EPR spin trapping is also in good agreement with the NO_x_ removal efficiency. Bulk C_3_N_4_ (CN) shows the lowest NO_x_ conversion in accordance with the lowest concentration of ^∙^DMPO-O_2_/O_2_H. On the other hand, the S-doped exfoliated g-C_3_N_4_ (Mes-Ex-CN) exhibits the highest NO_x_ conversion and the highest concentration of ^∙^DMPO-O_2_/O_2_H. The irradiation of pure TiO_2_ under identical experimental conditions (DMPO, water, air, UV light) resulted in the generation of the ^∙^DMPO-OH spin-adduct only, as published in previous work [24] confirming the dominant role of photogenerated hydroxyl radicals for this metal oxide photocatalyst.

As described in [5], the superoxide radical is able to oxidize NO to NO_3_^−^ (Equation (11)). Another possibility is the formation of hydrogen peroxide (Equation (12)) which is then further reduced to HO^∙^ radicals (Equation (13)) [25]. During photocatalytic removal of NO, the production of a typical by-product, NO_2_, was observed. NO_2_ is formed most probably, as in the case of TiO_2_, by its reaction with nitric acid (Equation (8)). Production of NO_2_ was observed for both UV and VIS irradiation and its production was proportional to the conversion of NO. In the case of photocatalysts, which exhibit lower conversion of NO, production of NO_2_ was lower (CN, S-CN). On the other hand, in the case of samples where the conversion of NO was significantly higher (TiO_2,_ Ex-CN, Mes-Ex-CN), we observed a higher production rate of NO_2_. In the case of TiO_2_, the surface area had no significant effect on NO_2_ production. The production rate in TiO_2_ CG 100 (crystal size 12 nm, S _BET_ 70–110 m^2^/g) is almost the same as in the case of P25, which exhibits a slightly lower surface area (45 m^2^/g, crystal size 22 nm). Interesting is the comparison of NO_2_ formation and overall NO_x_ removal, respectively, on CG100 and Mes-Ex-CN under UV irradiation. CG100 exhibited a slightly higher BET surface area compared to the S-doped exfoliated g-C_3_N4 sample (67 m^2^/g). The removed amount of NO was similar, but in the g-C_3_N_4_ sample the amount of produced of NO_2_ was lower, which influenced the NO_x_ overall removal rate. It seems that in the case of g-C_3_N_4_, the direct oxidation of initial NO to nitrate (Equation (11)) is the predominant pathway of NO removal, while two step oxidation of NO to NO_3_^−^ (Equations (6) and (7)) is more typical for TiO_2_ samples.

O_2_ + e^−^ → O_2_^∙–^ (10)

NO + O_2_^∙–^ → NO_3_^−^ (11)

O_2_^∙–^ + 2H^+^ + e^−^ → H_2_O_2_ (12)

H_2_O_2_ + e^−^ → HO^−^ + HO^∙^ (13)

## 4. Conclusions

Synthesized exfoliated and S-doped g-C_3_N_4_ photocatalysts were characterized and the photocatalytic removal of air pollutants (acetaldehyde and NO_x_) was evaluated according to the ISO 22197 and ISO 22197-1 methodology. Photocatalytic conversion of acetaldehyde was negligible for g-C_3_N_4_ materials. This can be explained by the valence-band edge position of the g-C_3_N_4_ photocatalyst, as the photogenerated holes are not able to generate hydroxyl radicals, which are considered to act as a primary oxidation species in acetaldehyde removal.

In contrast, in the case of g-C_3_N_4_, the most significant photoinduced species is the superoxide radical anion (O_2_^∙^^−^), as proved by the EPR spin-trapping technique. S-doped exfoliated g-C_3_N_4_ showed comparable NO and higher NO_x_ removal as TiO_2_ under UV irradiation. Besides, both exfoliated and S-doped exfoliated g-C_3_N_4_ showed very promising NO_x_ removal efficiencies under VIS irradiation. The pathway of NO removal on g-C_3_N_4_ was different from that on TiO_2_, as direct oxidation of NO to NO_3_^−^ by the superoxide radical anions played a major role. Formation of NO_2_ was thus diminished, which resulted in an improved total NO_x_ removal efficiency.

g-C_3_N_4_ exhibited a very high NO_x_ removal efficiency not only under UV irradiation (which was comparable with that of TiO_2_), but also under visible irradiation.

## Figures and Tables

**Table 1:** Summary and properties of studied photocatalytic materials.

Sample Name	Preparation	BET Surface Area (m^2^/g)	Phase Composition (XRD, XRF)
TiO_2_ P25	(Producer: Evonik), flame hydrolysis of TiCl_4_	45	70% anatase 30% rutile
CG100	(Producer: Precheza), sulphate process	70–110 *	100% anatase
CN	Thermal treatment of melamine	11 **	—
Ex-CN	Thermal treatment of melamine following thermal exfoliation	90 **	—
S-CN	Thermal treatment of thiourea	20 **	Sulphur content XRF 0.22 (weight %) **
Mes-Ex-CN	Ex-CN with modification using methanesulfonyl	67 **	Sulphur content XRF 0.56 (weight %) **

* Values provided by producer, ** Values published in previous work [8].

**Table 2:** Experimental conditions of performed ISO-tests.

Standard	ISO 22197-1	ISO 22197-2
pollutant	NO_x_	acetaldehyde
inlet concentration (ppm)	1	5
flow rate (dm^3^/min)	3	1
UV irradiance (mW/cm^2^)	1	1
analysis	chemiluminescence analyzer	GC

## Supplementary Materials

The following are available online at https://www.mdpi.com/1996-1944/13/13/3038/s1, Figure S1: FTIR spectra of CN, Ex-CN and Mes.Ex-CN materials, Figure S2: XPS spectra of Me-Ex-CN material.
