# Supplementary material for: Graphitic Carbon Nitride for Photocatalytic Air Treatment
Source: Materials (Basel). 2020 Jul 7;13(13):3038. doi: 10.3390/ma13133038 (PMC7372426; doi:10.3390/ma13133038)
Supplement: Supplementary file 1 [file materials-13-03038-s001.pdf]

materials

Supplementary Information

Graphitic Carbon Nitride for Photocatalytic Air Treatment

Michal Baudys [1], Šárka Paušová [1], Petr Praus [2], Vlasta Brezová [3], Dana Dvoranová [3], Zuzana Barbieriková [3] and Josef Krýsa [1,*]

[1] Department of Inorganic Technology, University of Chemistry and Technology Prague, Technická 5, 166 28 Prague 6, Czech Republic; baudysm@vscht.cz (M.B.); sarka.pausova@vscht.cz (Š.P.)

[2] Institute of Environmental Technology, VŠB-Technical University of Ostrava, 17. listopadu 2172/15, 708 00 Ostrava-Poruba, Czech Republic; petr.praus@vsb.cz

[3] Institute of Physical Chemistry and Chemical Physics, Faculty of Chemical and Food Technology, Slovak University of Technology in Bratislava, Radlinského 9, SK-812 37 Bratislava, Slovak Republic; vlasta.brezova@stuba.sk (V.B.); dana.dvoranova@stuba.sk (D.D.); zuzana.barbierikova@stuba.sk (Z.B.)

* Correspondence: josef.krysa@vscht.cz

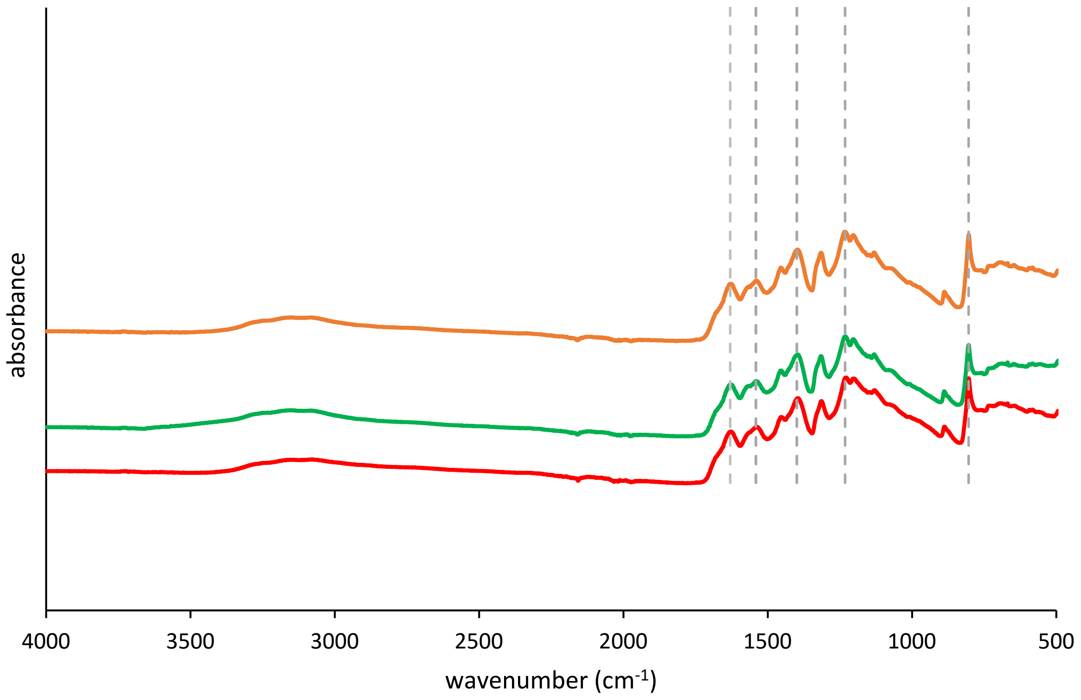

Figure S1. FTIR spectra of CN, Ex-CN and Mes.Ex-CN materials.

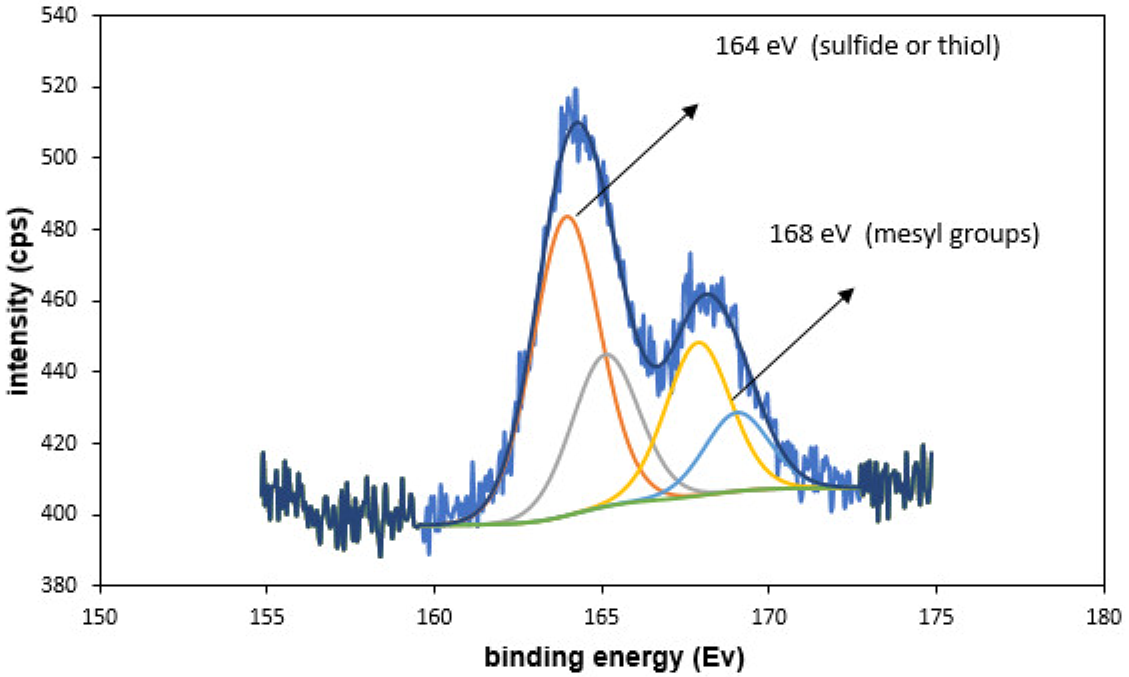

Figure S2. XPS spectra of Me-Ex-CN material.
